# Sensory Reinforcement Feedback Using Movement-Controlled Smartphone App Facilitates Movement in Infants with Neurodevelopmental Disorders: A Pilot Study

**DOI:** 10.3390/s26020554

**Published:** 2026-01-14

**Authors:** Anina Ritterband-Rosenbaum, Jens Bo Nielsen, Mikkel Damgaard Justiniano

**Affiliations:** 1 Elsass Foundation, Holmegaardsvej 28, 2920 Charlottenlund, Denmark; arr@elsassfonden.dk; 2 Department of Neuroscience, Panum Institute, University of Copenhagen, Blegdamsvej 3, 2200 Copenhagen, Denmark; jbnielsen@sund.ku.dk

**Keywords:** wearable technology, movement sensor, feedback, sensorimotor intervention, interactive, cerebral palsy, positive reinforcement

## Abstract

New wearable technology opens new possibilities for low-cost, easily accessible home-based interventions as a supplement to typical clinical rehabilitation therapy. In this pilot study, we tested a new interactive adjustable Feedback training system on 14 infants at high risk of cerebral palsy between 2 and 12 months of age to facilitate increased movements. The system consists of four wireless motion sensors placed on the infant’s limbs. Inertial sensors track the infant’s movements which control auditory and visual stimuli that act as motivational feedback. A 15 min usage of the Feedback training system four days a week for approximately six months was aimed for. None of the participants reached the recommended amount of intervention, due to time limitations. Seven of the twelve participating infants (58%) achieved at least 50% of the recommended training amount. Parents found the Feedback training system easy to use with minimal need for technical assistance. Preliminary data suggest that infants engaged more actively during training sessions where their movements actively controlled the presentation of the stimuli. The Feedback training system is promising as a user-friendly add-on to the playful and interactive stimulation of motor and cognitive development in infants with neurodevelopmental disorders.

## 1. Introduction

Cerebral palsy (CP) has a prevalence of two in every one thousand live births. The diagnosis designates a group of permanent disorders of the development of movement and posture, which causes activity limitations. These disorders are attributed to non-progressive disturbances that occur in the developing fetal or infant brain and are often accompanied by other conditions such as disturbances of sensation, perception, cognition, communication, and behavior, epilepsy, and secondary musculoskeletal problems [1]. Cognitive motor training, which stimulates sensorimotor development during infancy or early childhood, is likely important to prevent these secondary complications [2]. It has been argued that the period immediately after birth is a sensitive period—or a window of opportunity—where neuroplasticity seems to be most potent due to axons and dendrites sprouting while synapses are formed [3]. Animal studies have shown that the early nervous system undergoes maturation in an activity-dependent manner [4,5]. This appears also to be the case in human infants, as suggested from observations of alterations in the functional connections between cortex and spinal motor neurons around 3–4 months after birth [2].

This raises the possibility that children with neurodevelopmental disorders would benefit from goal-directed, functional training that can stimulate the infant to engage actively with their surroundings at this early time following birth. However, this is a challenge since verbal instructions and encouragement are difficult to employ and alternative ways of motivating and stimulating the infants therefore need to be applied. Interventions targeting early infancy which are based on self-initiated goal-directed movements are not a new idea. For example, Thelen and colleagues used a mobile reinforcement paradigm training system for infants at the age of 5–6 months. They suggested that infants would increase their activity when they were presented with positive feedback based on their own movements. This has been interpreted as improved sensorimotor comprehension when babies begin to predict the consequences of their (own) movements [6,7,8,9,10].

Improvement in motor outcomes is higher when children are engaged in training sessions in their natural environment/at home and when parents support their young child during training sessions [11,12].

To facilitate this, low-cost, easily accessible wearable technology that may monitor the movements of the infant and provide feedback for the infant, the parents, and therapists is now available [13,14].

With the use of the methodology from Translating Intention into Action (TIA) [15], we have completed a six-month home-based pilot study with a low-cost, easily accessible system, which can stimulate active movement in infants with a high risk of CP at the age of 3–9 months using reinforced positive feedback. The Feedback training system consists of (a) a standard tablet/app for auditory and visual stimulations, and (b) four small wireless inertial measure units (IMUs).

## 2. Materials and Methods

### 2.1. Ethics Approval

The local ethics committee, Denmark, Region H, granted approval for the study (H-19041562), and all parents provided written consent prior to participation in the intervention after verbal and written information about the project. All experimental procedures conformed with the latest revision of the Declaration of Helsinki. All personal data were anonymized. Names were assigned to an identification number, and data were stored according to the guidelines provided by The Danish Data Protection Agency.

### 2.2. Participants

We recruited 14 infants (mean age at start of intervention: 19 weeks, range: 14 to 32 weeks) from the neonatal and pediatrics departments in Denmark based on their medical records of high-risk status of CP. Infants were part of a larger intervention study following the CONTRACT protocol [16]. The inclusion criteria were as follows: (1) suspicion of brain lesion determined from a medical assessment, by MRI or ultrasound (US) scans, (2) absence of fidgety movements (FMs) as determined by General Movement Assessment (GMA). The brain lesion should be rated severe enough by the clinician to have informed the parents of the associated risk of CP. Absence of FMs has been shown to be highly predictive for CP especially in combination with MRI (95–98% [17]). Two infants dropped out due to personal reasons prior to the actual start of the intervention. We included clinical measurements defining the functional level of the infants. These tests were Hand Assessment of Infants (HAI) [18], Alberta Infant Motor Score (AIMS) [19], and Infant Motor Profile (IMP) [20] (see Table 1). For some infants, clinical tests could not be performed due to the infant’s inability to cooperate with the therapist or lack of time.

### 2.3. Technical Design

**Figure 1:**
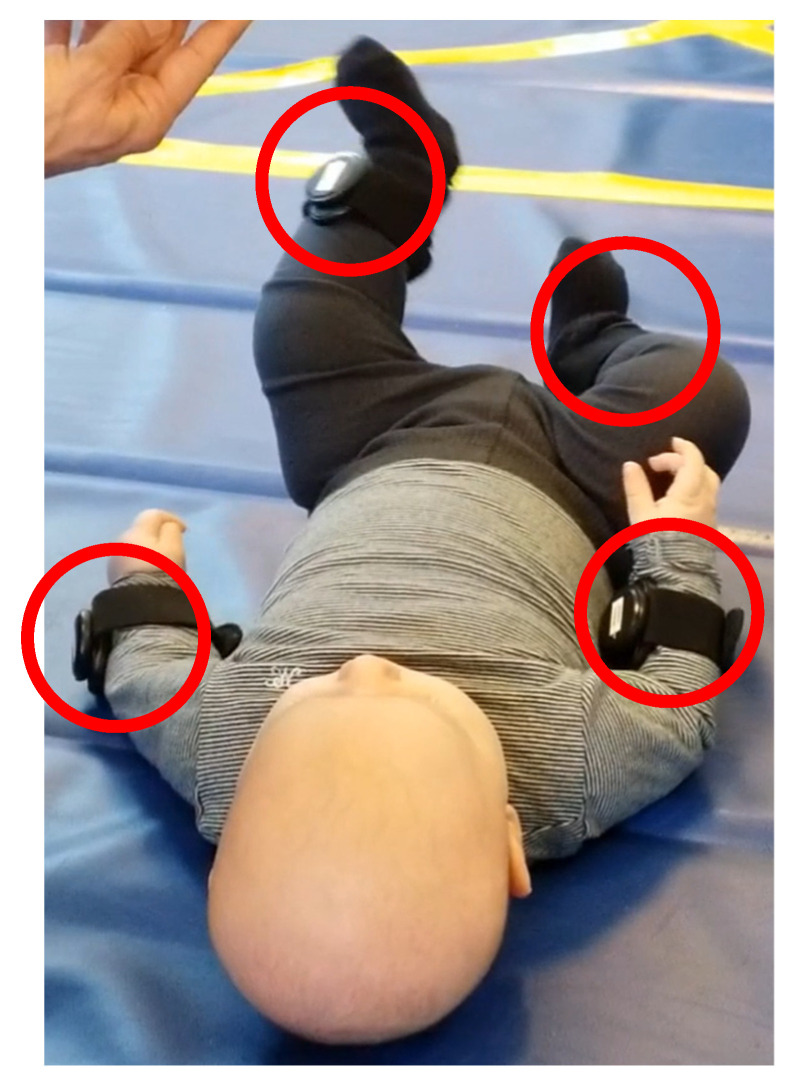
Illustration of the technical setup.

We developed the Feedback training system based on TIA [15] (https://github.com/Welfaretech-EF/TIA, accessed on 7 January 2026) as an interactive adjustable app connected to four wireless motion sensors, placed on the infant’s extremities (see Figure 1). The app adapted to the movements of the infant in order to motivate increased movement repertoire. The feedback training was tailored to the infants’ functional abilities and could be adjusted by the parents to present different videos/scenarios for the infant. These videos were chosen by the parents from a catalog of videos made specifically for infants, with high contrast and engaging music. The motion sensors were 9-axis IMUs (Movesense Sensor HR+, Movesense Ltd., Vantaa, Finland), recording linear acceleration, angular velocity, as well as the magnetic field at 26 Hz. The weight of each sensor was 20 g (including wristbands). For ease of use, each sensor automatically connected to the app on startup and the parents could start a session with a single touch on the tablet. The sessions consisted of active sessions, where the videos were controlled by the infants’ movements (angular velocity), and passive sessions, where the videos were played independently of the movements. These passive sessions were used to measure the effect of the infants’ engagement during the active sessions. The two types of sessions were presented randomly. For the sessions controlled by the infant’s movements, the angular velocity was used as input. To make the system adaptable to different levels of activity, an adaptive threshold was integrated instead of a fixed one. The adaptive threshold was based on a five second window of the latest activity signal for each sensor. This threshold was calculated as an equally weighted sum of the maximum and average activity during the last five seconds, corrected with respect to a fixed background offset in the sensor signal. The formula is presented in the following.

$$\tau \left(t_{0}\right)=\frac{1}{2}\left(\underset{t\in \left[t_{0}-5,\;t_{0}\right]}{\max}s\left(t\right)+\frac{1}{5}\int_{t_{0}-5}^{t_{0}}s\left(t\right)\;dt\right)+\varepsilon$$

Here, $t_{0}$ is the current time in seconds, $s\left(t\right)$ is the signal at time *t*, and ε is the background noise (the signal when no movement is occurring).

This adaptive approach ensured a gradual presentation of the stimuli to prevent abrupt changes. Preliminary testing of the adaptive threshold yielded a good balance between being inclusive to all activity levels and at the same time requiring an increase in the activity level. All movement data from the different sessions were stored locally on the tablet, until connection to the internet, which allowed data to be sent to a secure server for monitoring the infant’s progress and later analysis. To see a demo of the system, see Supplementary Material. The source code for the app is available as open-source on Github: https://github.com/mdolsen88/Feedback (accessed on 7 January 2026).

Figure 1 illustrates the location (red circles) of the four IMUs attached to the infants’ upper and lower extremities. Each sensor has an indication of the limb it is to be placed on to ease application for patents.

### 2.4. Experimental Procedure

#### 2.4.1. Intervention

Families were invited to the Elsass Foundation, Denmark, where they were informed about the intervention and instructed in how the equipment should be used when performing the home-based training.

Through a manual produced for the purpose, the parents could follow the steps of how to equip the infant with the movement sensors on the four limbs and how they should use the tablet for the presentation of the training. Parents were informed that if they experienced any technical difficulties with the system, they should contact the research team.

All parents were encouraged to train with their infants four times a week for 15 min during the intervention period. If they were unable to complete the daily training due to sickness, vacation, or other planning, they were encouraged to report this in an individual journal, which followed the family during the intervention. The journal was a simple date-based table, where the parents could indicate the amount of training each day and the reason for not performing the training.

The infant had to be awake, fed, and comfortable every time a training session started. If the infant was unhappy, the training should be postponed to a more ideal time. The infant was equipped with four sensors which were strapped on by a Velcro band on the distal parts of the four limbs. The straps were made sufficiently tight to ensure that the sensors would not be displaced when the infant was moving. The infant could be in a supine or sitting position to perform the training. The tablet was placed 30–50 cm away from the infant using a flexible tablet holder. Five consecutive sessions were presented on the tablet lasting three minutes each allowing pauses between the sessions if the infant needed that. The videos were controlled by the infant, by gradually starting and stopping the video in relation to the limb movements. Each session could be adjusted so one specific limb, two, or all limbs would be the main controller for the feedback training. For research purposes, we included sessions which corresponded to catch trials or so-called passive trials, where the video would play regardless of the movements of the infant. These sessions were used as basic levels for the infant’s movements. Parents were encouraged to help their infant throughout the training with verbal cues and pointing towards the screen, if the infant lost attention, but they should not touch the infant’s limbs.

#### 2.4.2. Questionnaire

After the intervention, the parents were asked to complete a short online questionnaire through SurveyMonkey (www.surveymonkey.com) containing 12 questions to report parents’ perspectives on the use of the Feedback training system for further development and use. The questions were related to three themes: (1) experience of how easy it was to be introduced to the Feedback training system and initiation of the home-based training, (2) parents’ experience of how their infant was engaged in the Feedback training system during the intervention period, and (3) how feasible parents considered the Feedback training system to be used as regular training. The first ten questions were to be evaluated based on a scale from 1 to 5 where the lowest number corresponded to the most negative experience/no and the highest number corresponded to the most positive experience/yes. The last two questions were to evaluate the time parents could see themselves using the equipment. See the template (in Danish) for the survey in Supplementary Materials.

### 2.5. Offline Analysis of Training Data

The data from the Feedback system were collected for each three-minute session. They contained information about whether it was an active or passive session, the duration and content of the session, as well as the movement data. All session data were saved, including those interrupted by the parents. Only complete sessions (three minutes consecutive data) were used for analysis.

A measure of overall activity was based on the median of the magnitude of the angular velocity for each session, combining the data from all four sensors.

Analysis of the effect of the Feedback system was only based on data from infants completing more than 50% of the planned intervention.

Data were also analyzed to detect a possible side difference between right and left (hand and foot data combined) to investigate if the app can detect a side difference. To ensure correct comparison between the right and left side, we excluded data from sessions where connectivity problems had occurred for at least one sensor. The calculation of side-difference was based on subtracting the left and right activity measures (based on the median of the angular velocity) and normalizing with respect to the combined sum. Positive values indicate a dominant right-hand side, and negative values indicate a dominant left-hand side.

### 2.6. Statistic

All statistical calculations which included correlation calculations, paired t-tests, and one-way/two-way ANOVA with Tukey post hoc test were performed in SigmaPlot version 14.5 (Alfasoft Limited, Np-105, Icentre Howard Way, Newport Pagnell, Milton Keynes, Buckinghamshire, MK16 9PY, UK). Some data are presented as averages for the group and some data are presented as individual data points. One standard deviation (SD) is used for the variation in data.

The level of significance was set at 0.05.

## 3. Results

**Figure 2:**
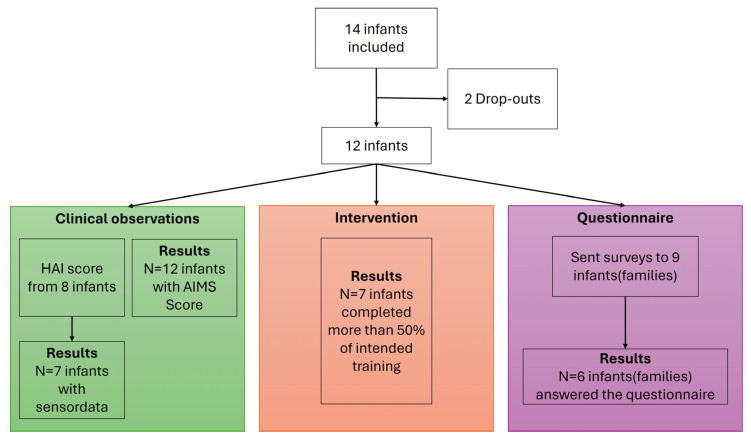
Flowchart of infants included in data analysis.

Data are presented within three different categories, namely intervention data based on sensor-based activity, the clinical observations combined with the sensor data, and finally the questionnaires. Figure 2 shows a flowchart describing how the initial 14 included infants are presented and used in the data analysis.

Figure 2 gives a graphical flowchart of the infants included in the analysis. Colors are only present to discriminate between the different types of data.

The time spent on the training varied greatly for the participants in terms of how many days they used the Feedback training system for and how many sessions/hours they completed throughout the intervention period (See Table 2). We found that seven of the infants achieved 50% or more of the recommended training. Out of the remaining seven infants, one family noted that this was not possible due to technical issues, while the remaining six families did not have time to do the training, in an already stressful period, despite regular contact with the research team.

Based on the seven infants included in the data analysis, we found that the infants trained for on average 13.3 (±1.8) minutes per training day.

**Figure 3:**
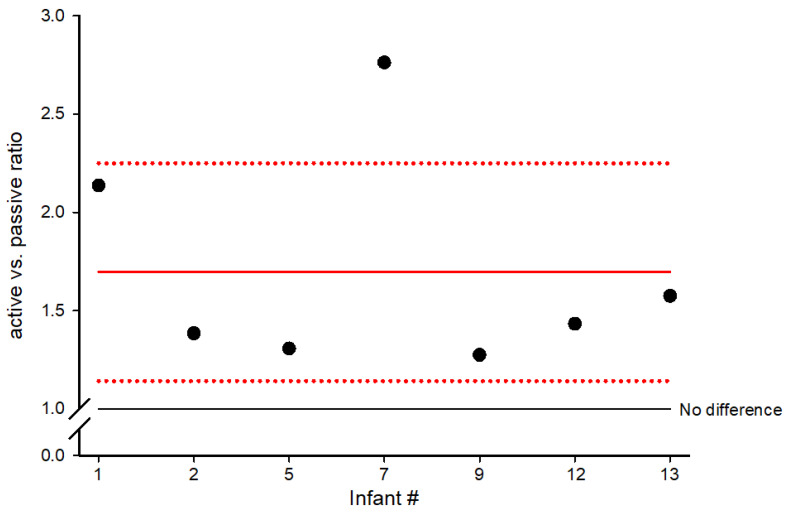
Active feedback increases activity level.

Figure 3 shows the ratio between the level of activity for passive and active sessions. In active sessions, videos were controlled by the infants’ movements (angular velocity) and in passive sessions the videos were played independently of the movement of the infants. The *x*-axis identifies the infants. The *y*-axis indicates the activity ratio between the active and the passive sessions during the training throughout the intervention period. The red line indicates the average active/passive ratio. Data above 1 on the *y*-axis indicate more activity during active sessions compared to passive sessions. Each data point is the average for each infant and one standard deviation is illustrated in the figure with red dotted lines.

Representation of active (infant-controlled feedback) and passive (videos playing independently) sessions covered on average 68% and 32%, respectively, of the total sessions. Infants significantly increased their activity level (paired *t*-test: *p* < 0.001), based on the median angular velocity for all active sensors, when they were presented with motor-controlled feedback (active sessions) during the training compared to when sessions were displayed on the tablet without their involvement (passive sessions). For graphical purposes (Figure 3), we have presented the ratio between the active sessions and the passive sessions for each training day. All data above 1 indicate more activity during the active sessions compared to the passive sessions.

We pooled all data to look at the side effects (right/left side upper extremities) and found a significant correlation between the clinical test of HAI based on each hand sum score and the Feedback training system data for recognizing the use of right vs. left hand. A linear regression analysis showed r^2^ to be 0.656 (*p* = 0.027, N = 7).

We included a correlation of the number of days trained for the infants and their score from the AIMS test. Linear regression analysis showed a non-significant negative correlation: r^2^:0.125 (*p* = 0.4, N = 12).

### Results from the Questionnaire

We sent out an online questionnaire to nine of the families included in the project. The answer rate was 60% and the average time to complete the questionnaire was approximately five minutes.

The average rating for questions about how to start the training and the parents’ need for additional technical support was 3.1 out of 5. Parents rated their overall impression of whether their infant liked the Feedback training system at 3.2 out of 5. The score for questions about whether parents would consider incorporating the Feedback training into their routine with their infant was 3.3 out of 5.

Four out of six families stated that 0–15 min of training with the Feedback system would be appropriate and reasonable for daily training. Two families found that 15–30 min would be appropriate for training. On average, we found that the families could consider training 3.3 ± 2.2 days/week.

**Figure 4:**
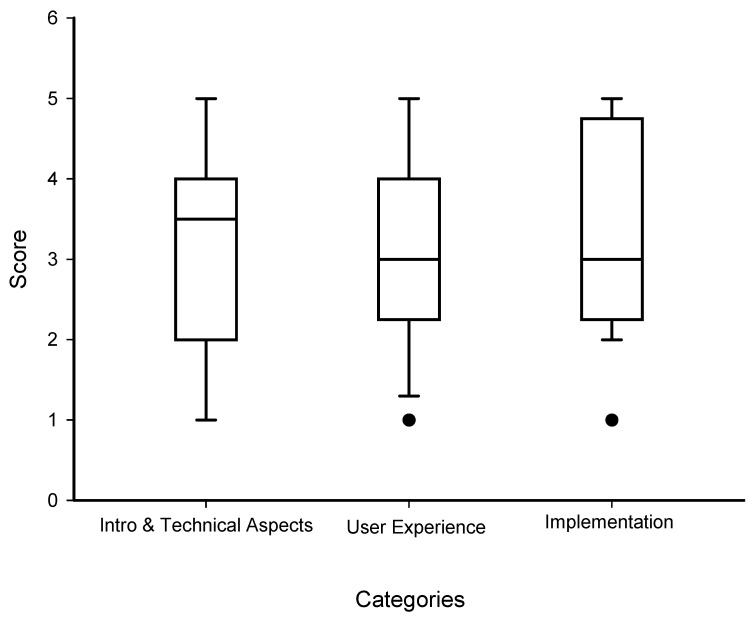
Parent experience questionnaire.

In Figure 4, the graph indicates the different categories of questions related to the parents’ experience of using the Feedback training system. Answers were rated between 1 and 5, where 5 was the most positive and 1 was the most negative experience. The median score is shown in the box plot (N = 6).

## 4. Discussion

The focus in this study was to evaluate the interactive Feedback training system as a novel supplement for clinical rehabilitation for infants in need of extra cognitive and motor training. Furthermore, we wanted to explore whether the Feedback training system is an effective way of encouraging, motivating, and maintaining more intensive focused home-based training than what is offered to infants at risk of CP. Most families reported a positive use of the Feedback training system. We reported two dropouts and three families who did not have time for the intervention during their daily life routine. However, the compliance of training was approximately 50% of what the study aimed for. We acknowledge that this participation is relatively low compared to other interactive home-based training systems, e.g., MiTii (Move it To improve it), which reported a training rate of 85% [21], or CareToy system, a home-based early intervention system, where authors reported compliance of 78% [22,23]. The studies mentioned had implemented routine communication with the family to maintain motivation and continuation of the training. This was scheduled about once a week [21,22,23,24]. In our intervention period, we had planned follow-up sessions with the families once a month. Justification for a reduced personal interaction (televised or physical meetings) is based on the fact that we wanted to create an add-on training system which could be available and implementable in the daily setting of families who are in need of extra training, but is limited in terms of support from healthcare providers due to limited resources [25,26].

The personalization of training is important, and the underlying heterogeneity of CP or other sensorimotor disabilities suggests that the strategies for treating an individual, and possibly monitoring or preventing further developmental complications, must be tailored to match that individual’s unique biopsychosocial profile [14,27]. This personalized rehabilitative intervention given by the functionality of the Feedback training system with the adaptive threshold which continuously monitors the level of the activity of the infant during training sessions can support and enhance usual care training possibilities to generate a precise and individually tailored intervention. Our data support the idea that instant positive feedback (active sessions where the infants controlled the feedback) makes the infants move their limbs more. The previous literature has already demonstrated that infants at the age of 2–4 months can couple their own movement with the sensory consequences (sound, light, touch) resulting in a positive loop which causes even more motor activity [7,28,29,30].

We see potential in the Feedback training system’s ability to identify subtle differences between the limbs. This offers an excellent opportunity for the system to serve as an additional identifier for infants who may later be diagnosed with hemiplegic or diplegic cerebral palsy by clinical staff. For implementation, we found that the Feedback system can be applied over an extended period to capture potential differences in activity levels between sides. The system can detect changes in motor patterns early on and provide supporting evidence for the infant’s motor and cognitive development, specifically targeting side differences [18]. A broad variety of training and therapy for babies exists, e.g., context-focused therapy, bimanual training, constraint-induced movement therapy (CIMT), neurodevelopmental treatment (NDT), goal-directed/functional training, muscle strengthening, and/or home programs for improving motor activities or self-care functions are some of the therapeutic approaches used in CP rehabilitation [31,32]. The interactive gaming technology, which this new app basically is, shows strength especially for the pediatric field as interactive interfaces can “hide” the fact that infants and children are training to obtain new motor and cognitive skills [27]. Severely affected infants may potentially benefit more from the Feedback training system as our data indicate a negative correlation between motor abilities and the amount of training the infants performed. Though it was a non-significant correlation, we consider that families where infants had more motor and cognitive abilities might need more challenging activities than “just” being placed in front of a tablet, to keep their attention on the training. Functional neurological changes have been argued to be present during this period [2], which might have an impact on the motor performance [33,34]. We acknowledge that one serious limitation of the project is the low number of infants in the study and caution should be taken in relation to the above-mentioned findings. Further exploration in a larger cohort is needed to validate the effect and possible impact of the system.

Our sensor-based Feedback training system was initially created to provide a home-based solution similar to the mobile device by Thelen and Fisher [35], by means of new technologies, such as TIA [15]. However, we found that despite the reported high acceptability of the Feedback training system, we recognize the difficulties or burden for the parents to interact with the technical aspects of the home-based system. One could argue that to create a solution with a higher impact, the solution should be integrated into the daily playtime routine with a limited technological interface to be adapted to the needs of the family, rather than the needs of the researchers. The literature indicates that a higher successful implementation of technology at home requires close collaboration between developers and end-users [36,37].

Technical solutions as add-on supplements for individual home-based intervention are available. It should be noted that such a solution would require Medical Device Regulation (MDR) approval, to ensure the correct measures for long-lasting personal usage at home. The MDR approval has not been explored, since the system was designed for clinical research purposes. Furthermore, a markerless system could be a possible solution to reduce preparation time, but issues regarding family privacy must be considered when introducing video-based motion capture at home. Easily available and portable markerless solutions have increased drastically mainly due to the rise in AI solutions and are already in clinical settings for analyzing infant motor behavior [38,39].

The current system is not completely ready for a stand-alone solution for home-based use as it requires preparation time for using the system as an integrated intervention session. Not all families have time in their busy daily life. However, from our results from the questionnaire, there is a positive attitude toward using the system. Another ideal solution could be to design a smart toy with built-in sensors that could identify and record the movements of the infants during activity and games in an interactive setting at home. This would reduce the technical preparations as the infant would not need to be equipped with wireless sensors to allow the technique to work. Even though the preparation is simple, it does require some time to set up, including ensuring that the battery and connection status is fine.

Another important element of the intervention is the amount of data being sent to the therapist and how much this should be fed back to adjust the intervention according to the infants’ needs and development. In the current set up, adjustments were made through the app where the parents had been instructed to make the adjustments, with support from the technical team.

When designing a tool for home-based intervention, it is important to balance the amount of responsibility put onto the parents and the need for contact and control from a therapist. Ideally, this add-on tool should be as independent as possible, making the system completely autonomous and adapt to the infants’ development. In a review identifying the possibilities and challenges with home-based technical solutions, it has been underlined that there is much flexibility within the intervention at home. However, care should be taken regarding the amount of technical expertise required in the setup and execution for both the families and clinical staff [14].

Modern technical solutions for improving home-based motor learning are not new ideas in the world of rehabilitation. During the COVID-19 pandemic, the use of telerehabilitation became an important tool for keeping the rehabilitation programs in place for the families [40,41]. Furthermore, with increasing demand for the healthcare systems [42], we need personalized home-based solutions to provide high-quality and sufficient intervention.

## 5. Conclusions

We conclude that the home-based Feedback training system can be implemented as an add-on training intervention for infants at the age of 3–10 months who are at risk of a developmental motor and cognitive disorder such as CP. The positive reinforcement in sound and visual feedback may stimulate increased activity, a finding that requires confirmation in a larger, controlled trial. The Feedback training system seems to be implementable in the homes of families with relatively little effort for all parties involved. It is crucial that the device is easy to use and accessible so it can be a part of regular care in a home-based setting. However, great attention is needed when introducing new technologies at home including clear and regular follow-ups for successful implementation. Though the data are very limited, the Feedback training system has potential for objectively identifying side differences in infants with CP and thereby initiating specific training at an early stage.

## Figures and Tables

**Table 1:** Describes the recruited infants including clinical scores.

ID	Corrected Age (Weeks at Intervention Start)	Dominant Hand Based on HAI Score (L/R)	AIMS
1	18	R	9
2	23	L	20
3	20	N/A	14
4	Dropout	N/A	N/A
5	18	No dominant hand	6
6	Dropout	N/A	N/A
7	17	N/A	9
8	14	N/A	8
9	32	N/A	10
10	17	L	17
11	14	L	11
12	21	R	11
13	14	R	9
14	22	R	27

Table 1 provides clinical information about the individual infants recruited in the study. The dominant hand is scored by left (L), right (R), or No dominant hand when scores did not detect a side difference with the Hand Assessment of Infants (HAI) test. N/A is indicated when tests are not applied. AIMS: Alberta Infant Motor Score.

**Table 2:** List of infants and days trained.

ID	Days Trained	Hours Trained	Questionnaires Sent (X) and Answered (✓)
1	93	22.6	X
2	57	12.1	X
3 *	0	0	X ✓
5	81	19	X ✓
7	57	8.6	X ✓
8 *	0	0	X ✓
9	93	22.2	X
10 *	8	1.7	
11 *	23	5.1	X ✓
12	88	21.2	X ✓
13	54	12.3	
14 *	0	0	

This table indicates the individual infants and how many days and hours they trained for during the intervention period. Infants with an asterix (*) did not complete the 50% intervention criteria.

## Data Availability

Data from the study are available on demand and all software codes can be found at https://github.com/mdolsen88/Feedback.

## Supplementary Materials

The following supporting information can be downloaded at https://www.mdpi.com/article/10.3390/s26020554/s1, Video S1: Early intervention with an adjustable interactive Feedback training system—a pilot study. Survey S1: Parent experience questionnaire.
