# Supplementary material for: Sensory Reinforcement Feedback Using Movement-Controlled Smartphone App Facilitates Movement in Infants with Neurodevelopmental Disorders: A Pilot Study
Source: Sensors (Basel). 2026 Jan 14;26(2):554. doi: 10.3390/s26020554 (PMC12845570; doi:10.3390/s26020554)
Supplement: Supplementary file 1 [file sensors-26-00554-s001.zip › Survey S1 SurveyMonkey_507030046.pdf]

Evaluering af Feedback-systemet - Forældre spørgeskema

Kære forældre,

Vi er igang med at evaluere brugen af Feedback-systemet, som I anvendte i forbindelse med interventionen i forskningsprojektet INCREASE i Elsass Fonden.

Vi håber at I har tid til at besvare dette korte spørgeskema som tager ca. 5 min at udfylde.
Jeres svar vil blive behandlet fortroligt og anonymt, og får ingen indflydelse på jeres videre forløb i projektet.

Tak for jeres deltagelse.
Mvh.
Feedback-systems teamet (Mikkel og Anina)
Elsass Fonden

1. Vælg det, der bedst matcher jeres oplevelse ved brugen af Feedback-systemet.

	Absolut Ikke	Nej	Måske	Ja	Helt sikkert
Virkede Feedback-træningen som en overskuelig indsats da I fik instruktionen omkring brugen af systemet?	○	○	○	○	○
Husk tilbage på første gang I selv skulle starte systemet derhjemme i forbindelse med interventionen. Var det nemt at komme igang?	○	○	○	○	○
Oplevede I at der var mange tekniske udfordringer ved brugen af Feedback træningen?	○	○	○	○	○
Gjorde I brug af kontakt til teknisk support undervejs i interventionsperioden?	○	○	○	○	○
Oplevede I at jeres barn kunne lide at bruge Feedback-systemet?	○	○	○	○	○
Oplever I at Feedback træningen har haft positive resultater på jeres barns udvikling?	○	○	○	○	○
Synes I at Feedback-systemet er egnet til hjemmetræning evt. som supplement til anden indsats?	○	○	○	○	○
Kunne I forestille jer at fortsætte Feedback træningen, hvis I fik muligheden?	○	○	○	○	○
Kunne I forestille jer at Feedback træningen bruges i længere tid hver dag end de 15 minutter som I blev instrueret i?	○	○	○	○	○
Kunne I forestille jer at anvende Feedback-systemet (sensorer + app) med jeres barn, hvis I selv skulle investere i udstyret?	○	○	○	○	○

2. Hvor mange minutter om dagen finder I det overkommeligt at gennemføre Feedback træningen?

- 0-15 min
- 15-30 min
- 30-60 min
- 60+ min

3. Hvor mange gange om ugen virker det overkommeligt at Feedback træningen skal udføres?

- 1 gang
- 2-4 gange
- 5-7 gange

4. Hvis I har andre kommentarer, som uddyber jeres svar eller som ikke er blevet adresseret i spørgeskemaet, kan I beskrive dem her:
